# Supplementary material for: The interactome of intact mitochondria by cross-linking mass spectrometry provides evidence for coexisting respiratory supercomplexes
Source: Mol Cell Proteomics. 2017 Dec 8;17(2):216–32. doi: 10.1074/mcp.RA117.000470 (PMC5795388; doi:10.1074/mcp.RA117.000470)
Supplement: Supplemental Data [file supp_RA117.000470_133922_0_supp_23978_fzffwf.zip › spectra_annotation/mito_DR_spectra_annotation/5-1-11-1-7-1.pdf]

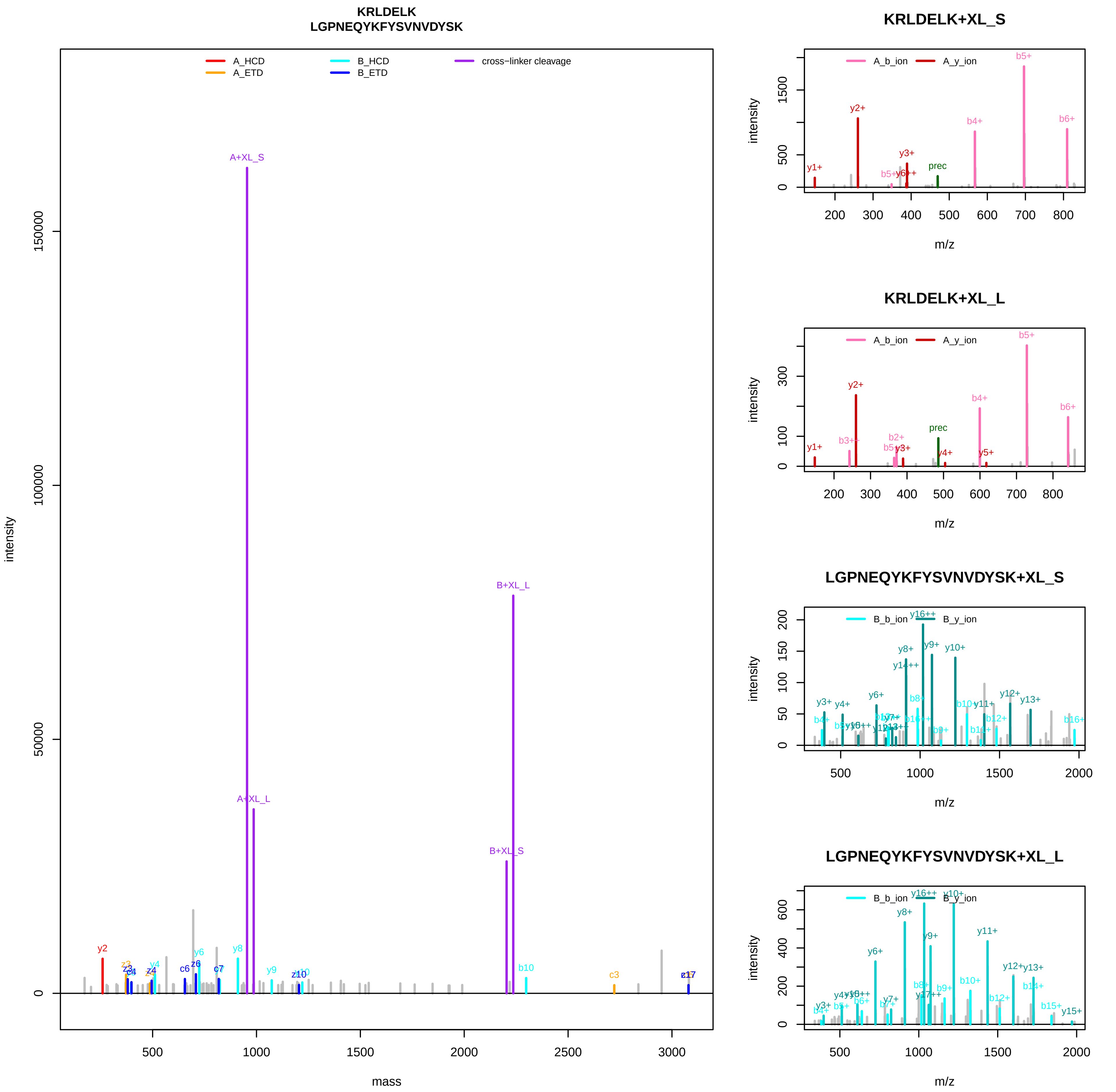
KRLDELK
LGPNEQYKFYSVNVDYSK
A_HCD
A_ETD
B_HCD
B_ETD
cross−linker cleavage
A+XL_S
A+XL_L
B+XL_L
B+XL_S
y2
z3
c4
z4
y4
c6
z6
y6
c7
y8
y9
z10
b10
c3
z17
intensity
mass
KRLDELK+XL_S
A_b_ion
A_y_ion
y1+
y2+
b5+
y6++
y3+
prec
b4+
b5+
b6+
m/z
KRLDELK+XL_L
b3++
b2+
y4+
y5+
LGPNEQYKFYSVNVDYSK+XL_S
B_b_ion
B_y_ion
y16++
y3+
y4+
y6+
y8+
y9+
y10+
y14++
b8+
y11+
y12+
y13+
b16+
LGPNEQYKFYSVNVDYSK+XL_L
y17++
b10+
b14+
b15+
y15+
